# ‘The good idea’: a commentary on a decade of barbed sutures in cesarean surgery

**DOI:** 10.1080/07853890.2026.2643495

**Published:** 2026-03-12

**Authors:** Maria Grazia Centurioni, Fabio Barra, Francesca Olcese, Claudio Gustavino, Simone Ferrero, Franco Alessandri

**Affiliations:** a Unit of Obstetrics and Gynecology, IRCCS Ospedale Policlinico San Martino, Genoa, Italy; b Department of Health Sciences (DISSAL), University of Genoa, Genoa, Italy; c Academic Unit of Obstetrics and Gynecology, IRCCS Ospedale Policlinico San Martino, Genoa, Italy; d Department of Neurosciences, Rehabilitation, Ophthalmology, Genetics, Maternal and Child Health (DiNOGMI), University of Genoa, Genoa, Italy

**Keywords:** Cesarean section, barbed suture, uterine scar, placenta accreta spectrum, uterine scar disorders, uterine niche, fish-bone suture, ultrasound

## Abstract

**Background:**

Minimalist cesarean techniques have delivered clear perioperative benefits, including shorter procedures, reduced tissue dissection, and reproducibility across diverse settings. As cesarean rates rise, however, an important blind spot has emergedhow the uterine scar evolves over months and years.

**Objective:**

To provide a narrative synthesis and methodological perspective on uterine scar healing after cesarean delivery, with particular attention to surgical determinants of lower uterine segment integrity and the emerging role of barbed sutures.

**Content:**

Post-cesarean niches and reduced residual myometrial thickness (RMT) are increasingly recognized as clinically relevant findings associated with abnormal bleeding, pelvic pain, subfertility, and difficult repeat surgery. Recently standardized ultrasonographic definitions allow these features to be measured reproducibly and compared across centers. Although evidence remains limited and heterogeneous, surgical factors influencing perfusion, tissue alignment, and tension distribution – shaped in part by suture material and closure technique – appear plausibly linked to long-term scar remodeling. Barbed sutures have gained interest because their knotless design distributes tension evenly and may help preserve perfusion and uniform coaptation. Comparative studies have reported associations between double-layer barbed closure and favorable imaging surrogates, such as thicker RMT and fewer or smaller niches, without apparent compromise in operative safety or efficiency. Evidence regarding subsequent pregnancy outcomes remains preliminary.

**Conclusions:**

Current signals regarding barbed sutures should be interpreted cautiously and underscore the need for methodologically rigorous investigations centered on uterine healing rather than operative speed alone. Future studies should incorporate standardized transvaginal ultrasound within one to two years after delivery, harmonized reporting of niche morphology and RMT, blinded image assessment, and predefined consideration of center and surgeon effects. Within this framework, double-layer barbed closure represents a plausible but provisional option whose clinical value requires confirmation through adequately powered multicenter research.

## KEY MESSAGES

- Uterine scar quality is an increasingly relevant clinical endpoint, as reduced residual myometrial thickness (RMT) and post-cesarean niches are consistently associated with abnormal uterine bleeding, pelvic pain, subfertility, and technical difficulty at repeat surgery.
- Uterine closure is not a neutral surgical step: the method and material of closure, through their effects on perfusion preservation, tissue alignment, and tension distribution, may influence long-term scar remodeling.
- Double-layer barbed uterine closure has been associated with more favorable imaging surrogates of healing, including thicker RMT and fewer or smaller niches; very preliminary, hypothesis-generating data suggest a possible association with greater third-trimester low uterine segment (LUS) thickness in subsequent pregnancies.
- The current evidence base remains limited and exploratory, highlighting the need for independent, standardized, multicenter studies incorporating post-cesarean ultrasound follow-up and harmonized reporting of niche morphology and RMT before any clinical implications can be drawn.

## Introduction

Cesarean delivery has been profoundly influenced over the last three decades by the spread of a minimalist philosophy – most clearly represented by the Misgav-Ladach/Stark technique – which reduced the number of surgical steps, favored blunt dissection, and made the operation reproducible across very different settings, from tertiary referral centers to low-resource hospitals. This shift translated into shorter operative times, lower postoperative analgesic requirements, and a higher degree of standardization of a life-saving procedure [1]. Its success, however, has also highlighted an aspect that remained relatively underexplored: the quality of uterine scar healing and its remodeling over the months and years following delivery.

This limitation has become more evident as cesarean rates continue to rise worldwide – from 21% in 2018 to projected rates exceeding 28%, and approaching 50% in some regions, by 2030 [2]. In this epidemiologic context, uterine scar disorders (also referred to as isthmocele or niches), technically challenging repeat cesarean deliveries with dense adhesions, and – in a subset of cases – placenta accreta spectrum (PAS) are encountered more frequently. If surgical quality is judged not only at ‘time zero’ (hemostasis, speed, uncomplicated recovery) but also by the structural and functional competence of the scar months or years later, then the phase of uterine closure warrants equal scientific attention [3,4]. At present, no universally supported strategy exists for preventing myometrial defects after cesarean delivery, as the literature on single- versus double-layer closure and locking versus non-locking techniques remains heterogeneous and sometimes conflicting [5,6]. Moreover, increasing evidence suggests that uterine closure strategies excluding the endometrial layer may reduce the prevalence of cesarean scar niches and potentially influence abnormal placental implantation [7–9]. Although techniques and study designs differ, this body of literature reinforces the concept that uterine closure is not a neutral step and that specific technical choices may have long-term structural and functional consequences [4].

Accordingly, an increasingly coherent concept is emerging: uterine scar healing – of which standardized imaging metrics such as niche characteristics and residual myometrial thickness (RMT) are important, though not exclusive, components – represents a clinically relevant endpoint after cesarean delivery that is sufficiently reproducible for use in comparative studies [6].

Against this background, interest has also grown in suture materials and closure devices designed to optimize tissue coaptation and tension distribution during hysterotomy repair. Among these, barbed sutures have been increasingly adopted in gynecologic surgery [10] and, more recently, in cesarean delivery, primarily because their knotless design facilitates continuous closure with potentially more uniform tension along the suture line. While their perioperative advantages have been relatively well described [11,12], their possible role in influencing uterine scar healing has only begun to be explored and remains incompletely defined.

While the clinical relevance of uterine scar healing and its imaging surrogates is increasingly well established, the relative contribution of specific surgical variables remains uncertain. In particular, the role of individual closure techniques and suture materials – including barbed sutures – should be regarded as an emerging hypothesis rather than settled evidence. As a commentary, this manuscript does not aim to provide a systematic or exhaustive review of the literature. Instead, the studies discussed are intentionally selected to illustrate key mechanistic and methodological aspects of uterine scar healing after cesarean delivery, with particular emphasis on investigations that assessed standardized imaging endpoints – such as niche morphology and RMT – or provided comparative data on uterine closure techniques. This selective approach reflects the heterogeneity and evolving nature of the field and is intended to frame hypotheses and research priorities rather than to summarize all available evidence.

## Discussion

Myometrial disruptions at the scar site are described in approximately 25–60% of women after cesarean, and their frequency seems to increase with the number of procedures: rates of 35%, 63%, 76%, and 88% have been reported after one, two, three, and four cesarean deliveries, respectively [13,14]. For years, the interpretation of these data was made difficult by the lack of uniform definitions. A dedicated modified Delphi process has substantially improved this situation by providing practical transvaginal ultrasound guidance for the non-pregnant uterus, recommending standardized assessment in both sagittal and transverse planes, measurement of the defect type (niche simplex or complex), and of the myometrium overlying and adjacent to it (RMT and adjacent myometrial thickness) [15]. Although these recommendations were conceived to make findings comparable across centers, they have been available only in recent years, so that the earlier literature still relies on non-uniform criteria. Even so, observational evidence has consistently linked lower RMT and larger niches with postmenstrual spotting, pelvic pain, subfertility mechanisms (blood pooling, mucus retention, altered uterine peristalsis), and procedural difficulties during embryo transfer for *in vitro* fertilization [16,17]. Very thin RMT, typically around 2.5–3.0 mm in non-pregnant assessments, is also used pragmatically to guide surgical repair and is often associated with higher symptom burden, even though thresholds that predict obstetric complications remain debated [16]. Taken together, these data do not prove a direct causal chain from scar defect to PAS or rupture, but they define an anatomically coherent, modifiable target for surgical quality improvement, consisting of a thicker and more homogeneous uterine scar on follow-up imaging.

Current knowledge suggests that cesarean scar disorders are multifactorial. They are favored by very low uterine incisions, by incomplete or technically suboptimal closure (for example, excessive suture traction and/or locking that compromises perfusion), and by impaired healing, particularly in the presence of adhesions between the scar and the bladder or abdominal wall [13]. Comparative and meta-analytic studies, despite heterogeneity of techniques and operators, tend to converge on the same signal: closures that avoid tissue strangulation and rebuild wall thickness – most notably a double layer with a non-locking suture – have been associated with greater RMT at 6–12 months [18,19]. In line with these concepts, closure strategies that deliberately exclude the endometrial layer have also been explored. In fact, some studies have reported that endometrial exclusion – whether achieved through endometrium-free closure techniques or by avoiding endometrial suturing – may be associated with a lower prevalence of cesarean scar niches [7–9]. In light of the above, the logical conclusion is that uterine closure should be engineered not only to stop bleeding quickly, but to preserve microvascular flow and to ensure uniform coaptation of the myometrial edges, creating the optimal biologic conditions for myometrial healing [6].

Within this framework, the rationale for barbed sutures appears both mechanistic and empirical. From a biomechanical standpoint, barbed sutures eliminate the need for knot stacks, which represent focal points of tensile load and may act as sites of localized tissue compression. By anchoring along the suture line, barbed devices are designed to distribute tension more evenly and to allow stable coaptation without the need for repeated tightening, particularly in an edematous and friable gravid myometrium. The intended effect is a continuous approximation with minimal dead space and potentially less focal ischemia. However, the extent to which these mechanical features translate into clinically meaningful reductions in ischemic injury depends on multiple interacting factors, including bite size, tissue selection, uterine thickness, degree of postpartum involution, and operator technique. Therefore, any potential benefit related to tension distribution should be interpreted as a contributory mechanism within a complex biological process, rather than as a standalone protective factor against adverse obstetric outcomes.

Empirically, gynecologic literature outside the cesarean field has documented the practical advantages of this approach. In laparoscopic myomectomy, more than 15 years ago, a randomized study comparing unidirectional barbed sutures with conventional continuous sutures and intracorporeal knots demonstrated shorter suturing time and reduced blood loss, without early safety penalties. This finding is relevant in a setting where procedural efficiency and technical simplification are important, while adequate uterine wall healing remains the ultimate objective [20]. Subsequent comparative cohorts extended this signal beyond intraoperative outcomes, reporting similar pregnancy and live-birth rates and no excess of adverse obstetric events after myomectomy performed with barbed versus non-barbed sutures [21,22]. The comparison with laparoscopic myomectomy provides a useful mechanistic analogy, insofar as both procedures require restoration of uterine wall integrity under conditions where excessive tension and ischemia may impair healing. However, important differences must be acknowledged. Myomectomy closure deliberately excludes the endometrium and occurs within a vascular environment that differs from that of cesarean hysterotomy. For this reason, evidence from myomectomy should be interpreted as supporting the general principle that tension-distributing suturing may favor healing, rather than as direct evidence transferable without modification to cesarean closure.

Two systematic reviews with meta-analysis, based on studies published in the previous ten years, have shown that barbed sutures are a suitable alternative to conventional materials for uterine closure at cesarean, reducing uterine repair time, total operative time, and the need for additional hemostatic stitches, without increasing blood loss or maternal morbidity [11,12]. Nonetheless, we believe these perioperative advantages, while real, should not be the unique focus of outcome assessment. What will shape future reproductive health is not whether the uterine layer was completed 3–4 min faster, but whether the uterine scar is thicker, the niche is absent or small, symptoms are fewer, and the uterine wall remains reassuring in the subsequent pregnancy. That is the perspective that informed our decade-long experience and that, in our view, justifies the title ‘The Good Idea’.

**Figure 1.**
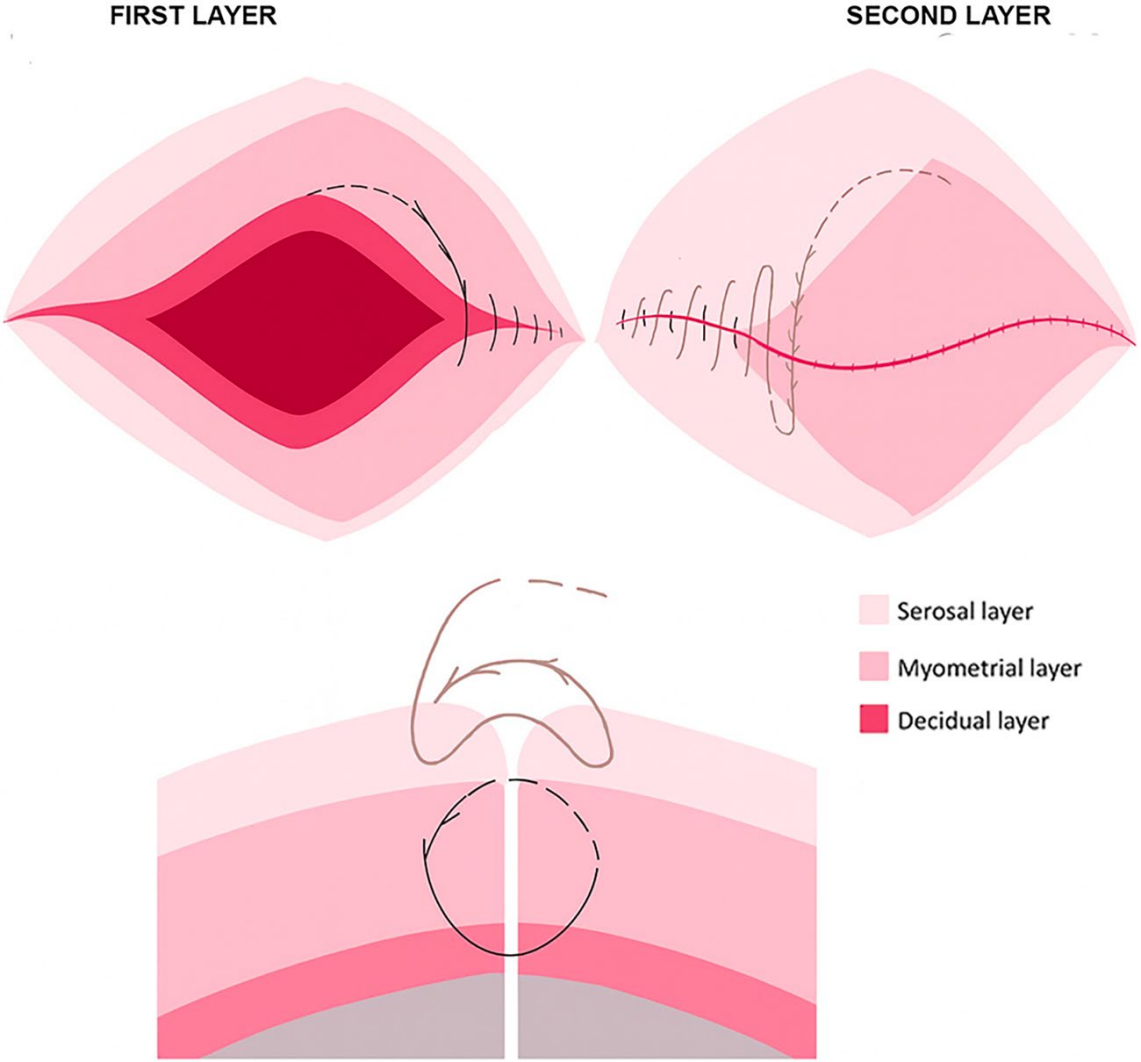
Schematic representation of double-layer ‘fish-bone’ suture. This figure is intended for illustrative purposes only, to depict the technical steps of the closure. It does not represent comparative outcome data and should not be interpreted as implying superiority over alternative closure techniques.

In our own single-center experience, we first described the ‘fish-bone’ technique (Figure 1), designed to secure the angles, run a continuous double layer, and avoid knot stacks in order to preserve perfusion and thickness in the most vulnerable isthmic region [23]. In a following prospective comparative study by our research group on primary cesarean at ≥37 weeks, 102 women whose uterus was closed with a double-layer unidirectional barbed (defined ‘fish-bone’) suture had fewer uterine niches at 6 months than those closed with a conventional double-layer smooth suture (20.2% vs 32.6%), and the defects, when present, were significantly shallower (*p* < 0.001). The barbed group maintained this advantage at 12 and 24 months, and women in the smooth group reported more postmenstrual spotting and more spotting days per month, confirming the concordance between imaging and symptoms [24]. These findings should be interpreted within the limits of a single-center design and a restricted number of surgeons, and therefore primarily as hypothesis-generating rather than definitive evidence of superiority. Nevertheless, this study, with predefined ultrasound timepoints, shifted the discussion from operating-room surrogates to the actual behavior of the scar over time.

A major merit of this approach is that it has now been independently reproduced. The Japanese multicenter randomized SPIRAL trial, which enrolled more than 200 women, found that at 6–7 months the barbed group had smaller niches (length 2.45 ± 1.65 vs 3.79 ± 1.84 mm; depth 1.78 ± 1.07 vs 2.70 ± 1.34 mm; width 1.58 ± 2.73 vs 2.88 ± 2.36 mm; all *p* < 0.001), a thicker RMT (8.46 ± 1.74 vs 7.07 ± 2.19 mm; *p* < 0.001), and, most importantly, a much lower proportion of women with a niche (29.1% vs 68.2%), with no RMT < 3 mm in the barbed arm – at comparable operative times and complication rates [25]. This confirms that the difference lies in healing, not in safety. A subsequent sub-analysis of the same cohort, however, showed that RMT was still significantly influenced by the center and by surgeon experience (*p* = 0.020 and *p* < 0.001, respectively), indicating that part of the ‘barbed effect’ is operator- and setting-dependent [26]. This is precisely why, in our study, all barbed closures were performed by two surgeons and all smooth closures by one surgeon: to maximize technique reproducibility and reduce variability attributable to individual practice [24].

Interestingly, the SPIRAL sub-analysis did not find a significant association between cervical dilation at cesarean and RMT (*p* = 0.215) [26]. This result contrasts with several observational series – and with day-to-day clinical experience – suggesting that advanced dilation, a laboring low uterine segment (LUS), and difficult extraction may produce thinner scars and more niches, probably through edema, overstretching, and irregular myometrial edges at the moment of repair [27]. Our working hypothesis is that, in these ‘unfavorable’ labor conditions, a tension-distributing barbed double layer might provide an additional protective effect by securing the angles and maintaining coaptation even when the segment is thinned and fragile. This is one of the areas we are currently exploring.

In the SPIRAL randomized trial [26], both barbed and conventional sutures were used in a double-layer closure that included the inner lining during first-layer repair, without deliberate endometrial exclusion. In contrast, in our previously reported series [24], the barbed ‘fish-bone’ technique was designed to limit decidual inclusion. The first layer incorporated only a minimal decidual thickness (<5 mm) together with the inner myometrium, while the second layer reconstructed the remaining myometrial wall and serosa. This distinction is relevant, as it suggests that favorable imaging outcomes associated with barbed sutures have been observed across different approaches to decidual handling, ranging from full inclusion to minimal inclusion. Rather than competing explanations, we deem that suture mechanics and tissue selection should be viewed as interacting determinants of healing. Importantly, although the SPIRAL trial confirmed more favorable healing-related imaging parameters in the barbed group [26], subsequent sub-analyses demonstrated that residual myometrial thickness was still significantly influenced by center and surgeon experience, indicating that suture material alone does not fully account for the observed differences [27].

A decade of experience with barbed suturing in cesarean surgery should not, however, be over-read. PAS is a clearly multifactorial condition – number of prior cesareans, placental location, interpregnancy interval, surgical planes, infection, and individual biologic vulnerability all play a role – and it would be naïve to claim that suture choice alone can reduce PAS incidence at population level [28,29]. Moreover, PAS remains relatively rare even in high-cesarean settings; showing a meaningful reduction in PAS tied to one surgical variable would require very large, long-term, carefully adjusted studies, which are rarely feasible in the surgical setting. Importantly, no single surgical variable – including suture material – can be expected to independently determine the risk of PAS or uterine rupture. For this reason, the uterine scar becomes the necessary surrogate: RMT at the thinnest point and standardized niche dimensions, measured by blinded operators at established follow-up times with Delphi-based ultrasonographic criteria, are the most proximate, reproducible markers of how well the scar has healed. If closure improves this local anatomy, it is biologically plausible – though currently unproven – that it may modify the substrate associated with abnormal placentation. Alongside imaging, trials should collect operative data, early morbidity, and patient-reported symptoms, because these are the outcomes that generate real-world consultations. Rare obstetric endpoints are better monitored through registries and large observational cohorts than through single underpowered randomized controlled trials.

A further, exploratory, and hypothesis-generating topic is the behavior of the LUS in a subsequent pregnancy. In fact, LUS thickness measurement by ultrasound has been used to evaluate the quality of the uterine scar after cesarean delivery and is associated with the presence of uterine scar defect [30] and risk of uterine rupture [31]. Nevertheless, LUS thickness also reflects the cumulative effect of multiple factors, including number of prior cesareans, labor characteristics, interpregnancy interval, placental implantation site, and individual healing response, and should not be interpreted as a direct surrogate for rupture risk attributable to closure technique alone.

Some authors showed that women whose index cesarean was closed with a double layer had, in the following pregnancy at 34–38 weeks, a slightly thicker LUS and were less often below the pragmatic 2.0-mm threshold used to stratify the risk of rupture or VBAC (Vaginal Birth After Cesarean) failure [32]. That study did not include barbed sutures. In a preliminary, single-center experience with a limited sample size, our group reported that in women who became pregnant again after an elective term cesarean, those whose hysterotomy had been closed with a double-layer barbed ‘fish-bone’ suture had a thicker third-trimester LUS than those closed with conventional monofilament (2.5 ± 1.1 vs 2.1 ± 1.0 mm; *p* = 0.030), and a higher proportion of them remained above 2 mm (61.8% vs 39.0%; *p* = 0.034). After adjustment for placental site, gestational age at scan, and maternal characteristics, barbed closure remained independently associated with a thicker LUS (+0.49 mm; 95% CI 0.28–0.96; *p* = 0.038), with no rupture or PAS observed [33]. This is proof-of-concept, single-center evidence, and should be read as such; but it fills an important gap, because it shows that the anatomical advantage we document at 6–12 months can still be seen when the uterus is challenged by a third-trimester pregnancy. However, despite adjustment for selected variables, residual confounding related to labor characteristics, interpregnancy interval, placental implantation, and individual healing response cannot be excluded. In light of the above, we propose that LUS thickness in a pregnancy after cesarean section may be another explorative pregnancy-specific surrogate endpoint alongside 6–12-month niche/RMT in future trials.

Potential criticisms about the use of barbed suture for cesarean section still deserve to be addressed. A first, reasonable concern is the greater amount of foreign material left in an edematous gravid myometrium: one could argue that a fully continuous barbed suture, because it occupies the entire suture track, might, in theory, favor inflammation or infection. However, the available data have not shown higher rates of endometritis, puerperal fever, or readmission compared with conventional sutures, suggesting that any additional inflammatory burden is clinically limited [12]. Moreover, commonly used absorbable barbed monofilaments are designed to be substantially resorbed within approximately 90 days, limiting the duration of any potential foreign-body exposure.

A second point sometimes raised with barbed materials is the theoretical risk of bowel or omental entrapment on the barbs. This has been described in gynecologic laparoscopy [34], but to our knowledge, it has not been reported after cesarean uterine closure. Importantly, as a practical precaution to minimize any theoretical risk of visceral or omental snagging, the free suture tail should be trimmed flush with the serosa/myometrium, avoiding intraperitoneal exposure of barbs. Finally, the issue of cost remains. Barbed sutures are more expensive per unit, but unit price is not the same as value: if this type of closure results in a thicker scar, fewer and smaller niches, and fewer outpatient visits for postmenstrual spotting or pain, the overall economic balance may become favorable. This, however, should be demonstrated in randomized trials with an embedded health-economic component, rather than only inferred from theoretical assumptions.

It must be acknowledged that a substantial proportion of the available evidence on barbed sutures in cesarean uterine closure – including several studies discussed above – originates from a limited number of research groups, including our own. While independent data support the presence of a healing-related signal [25,26], the overall body of literature remains numerically limited. This concentration of evidence increases the risk of interpretive bias and limits generalizability, particularly concerning operator variability, center effects, and learning curves. For this reason, the present commentary does not advocate barbed sutures as a definitive standard but rather frames them as a testable implementation of broader biomechanical principles that warrant independent confirmation in methodologically rigorous, multicenter studies. Throughout this discussion, barbed sutures are therefore not proposed as a causal determinant of the prevention of PAS or uterine rupture, but as one modifiable technical element that may influence scar morphology within a multifactorial framework of risk.

## Conclusions

As cesarean rates rise, the healing of the uterine wall is increasingly recognized as a relevant indicator of surgical quality, extending the evaluation of cesarean delivery beyond perioperative efficiency to the determinants of tissue repair over time. Factors such as the level of incision, tissue handling, and the method and material of uterine closure may plausibly influence the morphology and function of the healed uterine scar. Nevertheless, the available evidence remains heterogeneous, and current observations should be interpreted within this context.

Barbed sutures may represent a closure strategy worthy of further investigation because their knotless design may facilitate more even tension distribution and uniform tissue coaptation. Existing comparative studies have reported associations with thicker RMT and fewer or smaller niches, without apparent compromise in operative safety or maternal morbidity [24,25]. However, these findings are exploratory and hypothesis-generating, and they do not support changes in standard clinical practice at present. Confirmation requires independent replication through adequately powered, multicenter studies explicitly designed to assess uterine healing as a primary endpoint.

Future research should adopt standardized and methodologically rigorous approaches, including transvaginal ultrasound assessment within one to two years after delivery, harmonized definitions of niche morphology and minimum RMT, blinded image interpretation, and prespecified consideration of center and surgeon effects. Outcomes should integrate anatomical parameters with patient-reported symptoms and, where feasible, include health-economic analyses. Rare obstetric outcomes – such as PAS and uterine rupture – are best addressed through large multicenter registries and long-term observational follow-up of subsequent pregnancies. In this perspective, barbed sutures should be regarded not as a solution, but as ‘The good idea’ worthy of careful testing within a framework focused on uterine healing.

## Data Availability

No datasets were generated or analyzed during the current study.
